# Transgenerational Diapause as an Avoidance Strategy against Bacterial Pathogens in *Caenorhabditis elegans*

**DOI:** 10.1128/mBio.01234-17

**Published:** 2017-10-10

**Authors:** M. Fernanda Palominos, Lidia Verdugo, Carolaing Gabaldon, Bernardo Pollak, Javiera Ortíz-Severín, Macarena A. Varas, Francisco P. Chávez, Andrea Calixto

**Affiliations:** a Centro de Genómica y Bioinformática, Facultad de Ciencias, Universidad Mayor, Santiago, Chile; b Centro Interdisciplinario de Neurociencia de Valparaíso (CINV), Facultad de Ciencias, Universidad de Valparaíso, Valparaíso, Chile; c Laboratorio de Microbiología de Sistemas, Departamento de Biología, Facultad de Ciencias, Universidad de Chile, Santiago, Chile; d Departamento de Biología Celular y Molecular, Facultad de Ciencias Biológicas Pontificia Universidad Católica de Chile, Santiago, Chile; University of Texas Health Science Center at Houston; New York University School of Medicine

**Keywords:** *Caenorhabditis elegans*, RNA interference, defense, diapause, pathogenesis, survival strategies

## Abstract

The dynamic response of organisms exposed to environmental pathogens determines their survival or demise, and the outcome of this interaction depends on the host’s susceptibility and pathogen-dependent virulence factors. The transmission of acquired information about the nature of a pathogen to progeny may ensure effective defensive strategies for the progeny’s survival in adverse environments. Environmental RNA interference (RNAi) is a systemic and heritable mechanism and has recently been linked to antibacterial and antifungal defenses in both plants and animals. Here, we report that the second generation of *Caenorhabditis elegans* living on pathogenic bacteria can avoid bacterial infection by entering diapause in an RNAi pathway-dependent mechanism. Furthermore, we demonstrate that the information encoding this survival strategy is transgenerationally transmitted to the progeny via the maternal germ line.

## IMPORTANCE

Bacteria vastly influence physiology and behavior, and yet, the specific mechanisms by which they cause behavioral changes in hosts are not known. We use *C. elegans* as a host and the bacteria they eat to understand how microbes trigger a behavioral change that helps animals to survive. We found that animals faced with an infection for two generations could enter a hibernationlike state, arresting development by forming dauer larvae. Dauers have closed mouths and effectively avoid infection. Animals accumulate information that is transgenerationally transmitted to the next generations to form dauers. This work gives insight on how bacteria communicate in noncanonical ways with their hosts, resulting in long-lasting effects providing survival strategies to the community.

## INTRODUCTION

*Caenorhabditis elegans* is exposed to a wide variety of bacterial species. Soil bacteria represent a diverse pool of pathogenic and nonpathogenic food, and the nematode’s responses to different bacteria can contribute to diverse effects on its survival (1). *C. elegans* has previously been used to identify virulence mechanisms of bacteria and to characterize host responses to infection (2). Host and microbe contribute to the outcome, and therefore, the magnitude of the host damage results from the host-microbe interaction, explaining why infection with a particular microbe can drive different effects on the same host (3).

Animals challenged with infectious microbes have three main options: to fight the microbial attack by activating physiological cellular defenses, a costly approach in terms of energy and self-damage, which includes the expression of antimicrobial peptides or mobilization of immune cells; to develop tolerance to the pathogen, which is the ability to maintain fitness in the face of infection (4); or to avoid any contact with the infectious agent (5). Compared to the defense strategy, the avoidance approach has the advantage of decreasing the risk of infection, as well as an energy-sparing effect.

Nematodes, like other organisms, can resist a large variety of stress conditions by entering diapause. Under conditions of starvation, increased temperature, or crowding, *C. elegans* worms enter diapause, becoming dauer larvae (6). Dauers have a specialized cuticle, their mouths are plugged internally, and they lack pharyngeal pumping (7). These characteristics allow dauers to survive many environmental insults. Pathogen infection is a major environmental threat for all organisms, and defense mechanisms are crucial for survival. The insulin/insulinlike growth factor-1 (IGF-1) signaling (IIS) pathway, a major regulator of stress responses, is involved in defense against pathogens and dauer formation (8, 9). Here, we describe the formation of dauers in the second generation of worms exposed to *Pseudomonas aeruginosa* and *Salmonella enterica* serovar Typhimurium strain MST1 as a strategy to avoid pathogen infection. We also show that the information to form dauers is transmitted only through the maternal germ line and is correlated with the translocation of the DAF-16 transcription factor to the nucleus. Finally, we demonstrate that this novel transgenerational survival strategy against bacterial pathogens is mediated by RNA interference (RNAi) effectors at multiple levels that are required for diapause formation in the second generation as an escape response to the infectious challenge.

## RESULTS

### *C. elegans* forms a diapause stage in the second generation as a response to infection by pathogens with moderate virulence.

**FIG 1:**
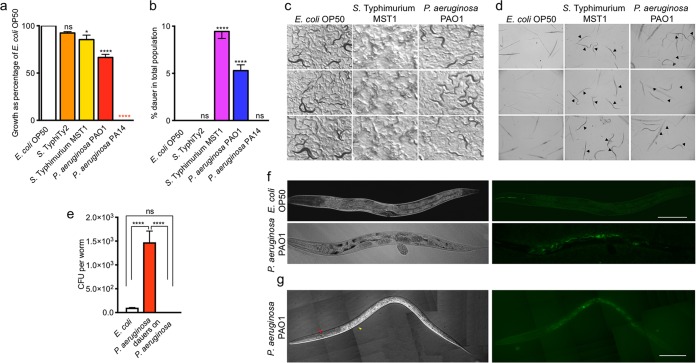
*C. elegans* enters diapause in the F2 generation as a defense against moderate pathogens. (a) Population growth on different pathogenic (*P. aeruginosa* PAO1, *S*. Typhimurium MST1, and *P. aeruginosa* PA14) and nonpathogenic (*E. coli* OP50 and *S*. Typhi Ty2) bacteria in the second generation. Population growth on all bacterial strains was normalized against population growth on *E. coli* OP50. (b) Dauer formation in the second generation of animals grown on different pathogenic and nonpathogenic bacteria. (c) Photographs of dauers on pathogenic and nonpathogenic bacterial lawns taken with a Sony XCD-SX910 camera on a trinocular SMZ745 stereomicroscope. (d) Photographs of animals treated with 1% SDS after growth on the three different bacteria. Pictures show a representative fraction of the 1% SDS drop. (e) Intestinal CFU of nondauer and dauer worms in the second generation growing on pathogenic and nonpathogenic bacteria. (f and g) Photographs of an F2 nondauer (f) animal fed with GFP-expressing *E. coli* or *P. aeruginosa* PAO1 and a dauer (g) animal fed with GFP-expressing *P. aeruginosa* PAO1. Scale bars represent 100 μm. Arrows show cuticular thickenings of dauers, ridges, or alae (red), and large striated zones along the body (yellow). ****, *P* < 0.0001; *, *P* < 0.05; ns, not significant. Error bars indicate standard errors of the means (SEM) of at least three biological replicas done in triplicates.

**FIG 2:**
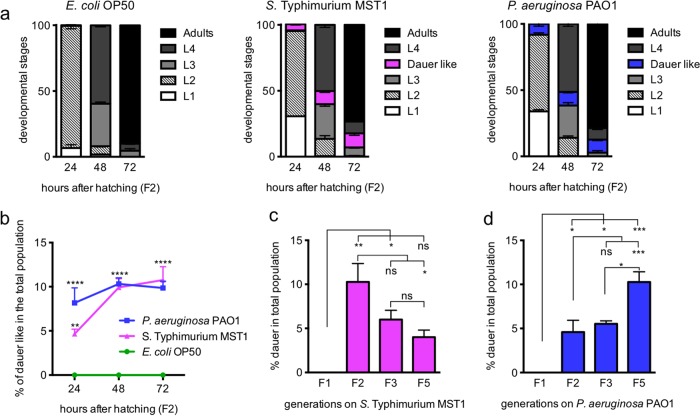
Development on pathogens. (a) Time course of developmental transitions and dauer formation on nonpathogenic and pathogenic foods. (b) Time course of appearance of dauerlike nematodes in the populations growing on pathogenic bacteria. (c, d) Dauer formation in successive generations on *P. aeruginosa* PAO1 (c) and *S*. Typhimurium MST1 (d). ****, *P* < 0.0001; ***, *P* < 0.001; **, *P* < 0.005; *, *P* < 0.05; ns, not significant. Error bars indicate SEM of at least three biological replicas done in triplicates.

The response of *C. elegans* to pathogens has been studied intensely (10–12). We are interested in studying the response of nematodes to pathogenic bacterium exposure in successive generations and the strategies used for long-term survival. To that end, we followed the growth of two generations of animals exposed to *S. enterica* serovar Typhimurium strain MST1, *S. enterica* serovar Typhi strain Ty2, *P. aeruginosa* strain PAO1, and *P. aeruginosa* strain PA14 and compared the growth of the total populations to their growth when exposed to *Escherichia coli* strain OP50, the usual laboratory food. We transferred 5 fourth-larval (L4)-stage parental hermaphrodites (P0), grown on *E. coli* OP50, to 90-mm nematode growth medium (NGM) plates seeded with large lawns of the different saturated bacterial cultures and followed the growth of the populations until the second generation (F2) in the same plate (see Materials and Methods). *S. enterica* serovar Typhi Ty2, a human-specific pathogen, did not affect the population number of *C. elegans*, while *P. aeruginosa* PAO1 decreased the population number by 30% and *S*. Typhimurium MST1 decreased it by 10%. *P. aeruginosa* PA14 killed worms in the first generation (Fig. 1a) (2). Interestingly, in the presence of *P. aeruginosa* PAO1 and *S*. Typhimurium MST1, the F2 progeny formed dauers, a nonfeeding larval stage specialized for survival and dispersal (Fig. 1b to d), while the F1 progeny did not (Fig. 2c and d). We quantified the amount of dauers formed on each bacterial strain by counting surviving larvae after treatment with 1% SDS (13). Totals of 5.3% and 9.4% of animals formed dauers on *P. aeruginosa* PAO1 and *S*. Typhimurium MST1 lawns, respectively. Worms undergoing starvation commit a similar number of animals to diapause and not the entire population (see Fig. S1A in the supplemental material), suggesting that under stressful stimuli of diverse natures, only a percentage of the population enters diapause and forms the dauer larvae. Importantly, dauers formed on the bacterial lawn (Fig. 1c; Movies S1 and S2) and not because of being starved outside the bacterial food area due to pathogen avoidance (14). Moreover, animals living on these pathogens eat normally, as shown by the rate of their pharyngeal pumping, which is even higher than on *E. coli* OP50 (Fig. S1B). To test whether dauers effectively avoid bacterial infection, we counted the number of CFUs in the intestines of nondauer F2 animals fed on *P. aeruginosa* PAO1 and compared them with the results for the intestines of F2 dauers. Animals growing on pathogens had significantly more bacteria in their intestines than those fed on *E. coli* OP50 (Fig. 1e). In contrast, bacteria were absent in dauer intestines (Fig. 1e). Additionally, for live imaging of intestinal bacteria in dauer and nondauer animals, green fluorescent protein (GFP)-tagged *E. coli* OP50 and *P. aeruginosa* PAO1 strains were observed under a fluorescence microscope. While the colonization of nondauer animals by *P. aeruginosa* PAO1 was readily observable (Fig. 1f), the intestines of dauers were devoid of fluorescence (Fig. 1g). These findings demonstrate that *C. elegans* diapause entry in the progeny of animals exposed to pathogens is a successful strategy to avoid bacterial infection in the absence of other sources of food.

10.1128/mBio.01234-17.1 FIG S1  Dauer formation under starvation and pharyngeal pumping rate on pathogens. (a) Five L4 animals were fed *E. coli* OP50 in a 60-mm plate and were allowed to grow for 10 days. Dauer formation in the second generation was assessed 2 days after food was completely exhausted. (b) Graphs illustrate the number of pharyngeal contractions in 1 min of animals feeding on the indicated bacteria for 72 h after hatching. ****, *P* < 0.0001; ***, *P* < 0.001; **, *P* < 0.005; *, *P* < 0.05. Bars indicate average results for 30 animals per plate in triplicates. Download FIG S1, TIF file, 2.8 MB. Copyright © 2017 Palominos et al. 2017 Palominos et al. This content is distributed under the terms of the Creative Commons Attribution 4.0 International license.

10.1128/mBio.01234-17.9 MOVIE S1  *C. elegans* culture of F2 animals feeding on *S*. Typhimurium MST1 prior to treatment with 1% SDS to quantify dauer larvae formation. Download MOVIE S1, MOV file, 11.9 MB. Copyright © 2017 Palominos et al. 2017 Palominos et al. This content is distributed under the terms of the Creative Commons Attribution 4.0 International license.

10.1128/mBio.01234-17.10 MOVIE S2 *C. elegans* culture of F2 animals feeding on *P. aeruginosa* PAO1 prior to treatment with 1% SDS to quantify dauer larvae formation. Download MOVIE S2, MOV file, 10.5 MB. Copyright © 2017 Palominos et al. 2017 Palominos et al. This content is distributed under the terms of the Creative Commons Attribution 4.0 International license.

### Pathogen-induced dauer formation in the second generation depends on bacterial virulence.

The outcome of an interaction between a pathogen and a host changes depending upon environmental and genetic factors (3). Because dauer formation was observed in populations exposed to pathogens of moderate virulence, we next explored whether the degree of virulence of the pathogen affects the number of animals entering diapause. We grew *P. aeruginosa* PAO1 in medium depleted of inorganic phosphate, which favors the expression of toxic pigments like pyoverdin and pyocyanin, which are virulence factors (Fig. S2A) (15). As previously reported (15), high-P_i_ conditions allowed wild-type worms to grow and reproduce on *P. aeruginosa* PAO1, while low P_i_ significantly decreased the viability of nematodes (Fig. S2B). Likewise, fewer dauer larvae were formed when fed with bacteria grown on low-P_i_ medium than with those grown on high-P_i_ medium (Fig. S2C). To further explore whether dauer formation under pathogenesis depends on the virulence of the pathogen, we tested the ability of avirulent *P. aeruginosa* PAO1 mutants with mutations of *lasR* (10), *pvdS* (15), and *ppk1* (16) to induce diapause. The results in Fig. S2D show that mutations in these genes severely affect dauer formation in *C. elegans*, and two of them (*lasR* and *ppk1*) reduced worm growth (Fig. S2E). This suggests that the worms’ ability to form dauers depends on the virulence of the pathogen. More virulent bacteria, like *P. aeruginosa* PA14 and low-P_i_-fed *P. aeruginosa* PAO1, as well as bacteria of low or no virulence like *E. coli* OP50 or avirulent mutants of *P. aeruginosa* PAO1, do not trigger this mechanism of defense. These results demonstrate that only pathogens of moderate virulence that can support host growth past the first generation but that sicken animals promote dauer formation.

10.1128/mBio.01234-17.2 FIG S2  Siderophore production of *P. aeruginosa* PAO1 under high- and low-P_i_ conditions. (A) Quantification of total siderophores, pyoverdine, and pyocyanin under high- and low-P_i_ conditions. Siderophore concentration was normalized as deferoxamine mesylate (Desferal) equivalent. (B, C) Population growth (B) and dauer formation (C) of animals on *P. aeruginosa* PAO1 under low-P_i_ conditions. (D, E) Population growth (D) and dauer formation (E) of animals on lawns of *P. aeruginosa* PAO1 mutants with decreased virulence. Each point in the graph represents the mean value of at least three experiments. ****, *P* < 0.0001; ***, *P* < 0.001; **, *P* < 0.005; *, *P* < 0.05. Error bars indicate SEM of at least three biological replicas done in triplicates. Download FIG S2, TIF file, 2.8 MB. Copyright © 2017 Palominos et al. 2017 Palominos et al. This content is distributed under the terms of the Creative Commons Attribution 4.0 International license.

### Developmental transitions and dauer formation on pathogens.

The decision to enter diapause occurs in the L1 stage when worms integrate several stress signals, which commit them to continue development or enter arrest as dauers (13). To assess the development and dauer formation of individual animals feeding on pathogens, we followed individuals every 24 h for 72 h and examined specifically whether worms with dauerlike appearance formed SDS-resistant larvae or exited the dauer program (Fig. 2a). Dauerlike animals (criteria explained in Materials and Methods) began to appear at 24 h only on pathogens (8.2% for animals on *P. aeruginosa* PAO1 and 4.7% for animals on *S*. Typhimurium MST1). At 48 h, 10% of animals exposed to either pathogen were dauerlike, a figure that remained constant at 72 h (Fig. 2b). On *S*. Typhimurium MST1, all dauerlike larvae became SDS-resistant dauers, while on *P. aeruginosa* PAO1, a smaller percentage (5.3%) of animals became SDS-resistant dauers, showing that a small percentage of animals feeding on *P. aeruginosa* PAO1 exited the initial dauer decision.

We wondered if the number of dauers in the population would increase or remain constant with every generation living on pathogens. To answer this question, we transferred for 5 generations fixed amounts of worms to new plates seeded with pathogens (40 L4 animals for *E. coli* OP50 and *S*. Typhimurium MST1 and 60 L4 animals for *P. aeruginosa* PAO1) and quantified the amount of dauers in every generation. Surprisingly, while on *P. aeruginosa* PAO1, the number of new dauers increased significantly from the F2 to the F5 (Fig. 2c), animals feeding on *S*. Typhimurium MST1 decreased their number of dauers in the F5 generation (Fig. 2d). This decrease could reflect the development of tolerance to the infection with *S*. Typhimurium MST1 with the passing generations, where dauer formation would no longer be needed to defend the population. *P. aeruginosa* PAO1, however, which expresses a plethora of virulence factors (17), is established as a *bona fide* pathogen, and therefore, the defense strategy is not only maintained but also reinforced throughout multiple generations.

### Dauer formation in response to pathogenesis requires persistent intestinal colonization.

**FIG 3:**
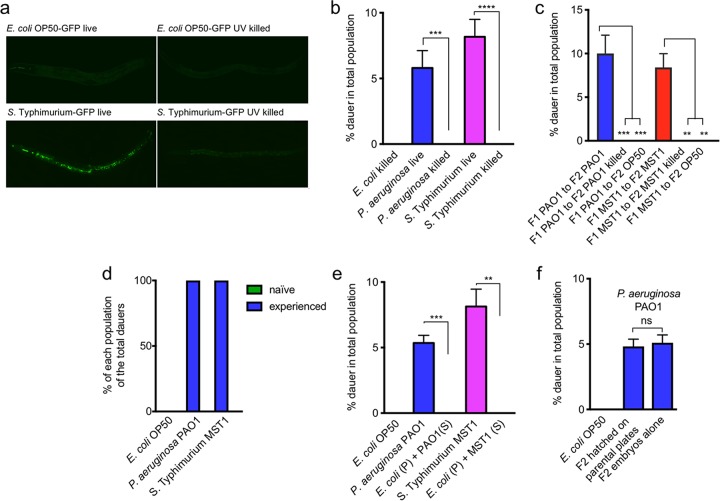
Bacteria need to colonize the intestine to induce diapause formation. (a) Photographs of live and UV-killed GFP-expressing *E. coli* OP50-GFP and *S*. Typhimurium MST1-GFP in worm intestines. Bacteria were killed by UV exposure for 20 min. Bars represent 100 μm. (b) Population growth and dauer formation of worms on live and UV-killed bacteria. (c) Dauer progeny of F1 animals grown on pathogenic bacteria were changed to either UV-killed pathogens or nonpathogenic *E. coli* OP50. (d) Dauer formation of animals with experience on the pathogen mixed with naive animals in the F2 generation. *P_mec-17_mec-17*::*gfp* animals (naive) as F2 embryos from hermaphrodites feeding on *E. coli* OP50 were mixed with a population of animals feeding on the pathogen for one generation (experienced). (e) Dauer formation of animals feeding on nonpathogenic *E. coli* OP50 supplemented with pathogen supernatant (S). (f) Population growth and dauer formation of F2 embryos hatched on plates devoid of parent animals compared with F2 dauer formation of animals in contact with their F1 parent animals. ****, *P* < 0.0001; ***, *P* < 0.001; **, *P* < 0.005; ns, not significant. Error bars indicate SEM of at least three biological replicas done in triplicates.

Pathogen and host mount mutual defensive strategies depending on the exchange of molecular signals in an active interkingdom communication (18, 19). To test whether pathogenic bacteria were required to colonize and actively communicate with their host to induce diapause, we fed worms with UV-killed *P. aeruginosa* PAO1 and *S*. Typhimurium MST1 and quantified their ability to induce dauer entry. UV effectively killed bacteria, since no GFP-expressing cells were visible in the animals’ intestines after the treatment (Fig. 3a and Materials and Methods). *C. elegans* grows (Fig. S3A) but does not form dauers (Fig. 3b) on UV-killed bacteria, suggesting that intestinal colonization of parental individuals is a requisite for dauer formation under pathogenesis. Additionally, when we changed F1 animals from live to UV-killed pathogens or to nonpathogenic *E. coli* OP50, their F2 generation did not form dauers (Fig. 3c). This shows that the second generation of animals only commits to diapause in the presence of metabolically active pathogens and that two generations on pathogens are needed for the dauer program to be expressed. Along the same line, we asked whether naive animals could be prematurely induced to form diapause in the F1 generation by being in contact with an experienced population of worms and bacteria for a shorter period. Gravid, nonfluorescent F1 worms living on pathogens were mixed with naive embryos expressing *gfp* (*P_mec-17_mec-17*::*gfp*), and after 3 days, all animals were treated with 1% SDS. One hundred percent of dauers were noncolored self-progeny of experienced hermaphrodites (Fig. 3d). This indicates that diapause formation requires persistent intestinal contact with pathogens for two generations and suggests that secreted molecules from experienced worms and pathogenic bacteria are not sufficient to induce diapause. To directly test whether secreted molecules from pathogens could induce diapause entry, we separated cells from supernatant of a saturated liquid culture of pathogens and supplemented a pellet of nonpathogenic *E. coli* OP50 with supernatant from *P. aeruginosa* PAO1 or *S*. Typhimurium MST1. As shown by the results in Fig. 3e, the pathogen’s supernatant is not sufficient to induce dauer formation in animals feeding on nonpathogenic *E. coli* OP50 (growth is shown in Fig. S3B). Finally, to test whether molecules secreted by two generations of worms exposed to the pathogen were required for dauer formation in the F2 generation, embryos from F1 hermaphrodites obtained by hypochlorite treatment were transferred to new plates with naive *P. aeruginosa* PAO1 (not previously exposed to worms). F2 animals born on plates bearing the pathogen and devoid of F1 parent worms formed the same amount of dauers as those in contact with their progenitors (Fig. 3f; Fig. S3C shows growth). These results show that direct interaction of bacterial cells with the worm intestine for two generations is needed for dauer formation, suggesting that the passage of information may occur through the germ line from infected parents to progeny.

10.1128/mBio.01234-17.3 FIG S3  Growth of bacteria under different bacterial conditions. Population growth of worms on live (A) and UV-killed (B) bacteria. S, nonpathogenic *E. coli* OP50 supplemented with pathogen supernatant. (C) Plates containing all animals for two generations compared to plates with embryos alone. ****, *P* < 0.0001; ***, *P* < 0.001; **, *P* < 0.005; *, *P* < 0.05. Download FIG S3, TIF file, 2.8 MB. Copyright © 2017 Palominos et al. 2017 Palominos et al. This content is distributed under the terms of the Creative Commons Attribution 4.0 International license.

### The information to form dauers is transmitted transgenerationally.

**FIG 4:**
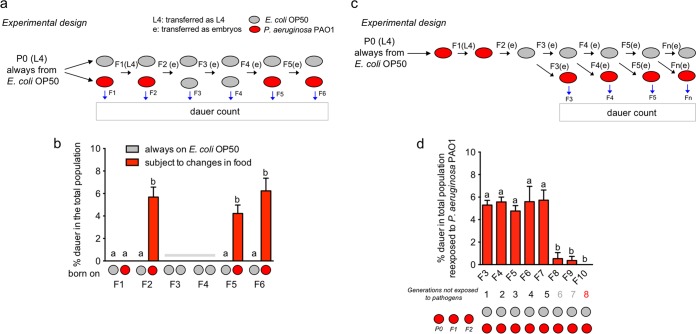
Dauer formation under pathogenesis is transgenerational. (a) Diagram of experimental design to determine transgenerationality. (b) Dauer formation of animals on pathogens after exposure for two generations on nonpathogenic bacteria. Nonpathogenic and pathogenic foods are represented by gray or red circles, respectively. Gray horizontal bar represents dauer counts not included in statistical analysis since all values were zero. (c) Diagram of experimental design to determine the number of generations the transgenerational effect is maintained. (d) Dauer formation of animals reexposed to pathogens after feeding for 8 generations on nonpathogenic bacteria. Error bars indicate SEM of at least three biological replicas done in triplicates. “a” and “b” are used to indicate statistically significant differences.

The passage of information about the pathogenic status of bacteria even in their absence could be advantageous for the progeny in mounting effective, quicker defensive strategies upon a new encounter with pathogens. We asked whether diapause formation under pathogenesis is transgenerationally transmitted to later generations. Transgenerational phenomena require germ line transmission of epigenetic information in the absence of direct environmental exposures (20). To test this, we exposed animals to *P. aeruginosa* PAO1 for two generations and changed them to *E. coli* OP50 in the F3 and F4 generations and later exposed them again to pathogens for two additional generations (Fig. 4a). As controls, we used animals growing on *E. coli* OP50 after every passage. To ensure that F2 animals did not carry traces of pathogens in their intestines or bodies, we treated adults with a hypochlorite solution in each generation and passed clean embryos to the next plate (Fig. 4a). Dauer formation was checked in every generation. As expected, neither F1 animals on pathogens nor animals on *E. coli* formed dauers. Importantly, F5 and F6 animals from parents that had skipped pathogens for two generations formed amounts of dauers similar to the amounts formed by the F2 animals (Fig. 4b), showing that the dauer formation strategy as a defense mechanism is transgenerationally transmitted. To determine for how many generations the transgenerational effect is maintained, F2 embryos were passed to nonpathogenic *E. coli* OP50, and for every subsequent generation, a fraction of the embryos was transferred to new *E. coli* OP50 plates and an identical fraction was transferred to *P. aeruginosa* PAO1 to check for the appearance of dauers (Fig. 4c and Materials and Methods). We observed similar percentages of dauer larvae from the F2 until the F7 generation, and then the percentage fell abruptly in the F8 generation (Fig. 4d). This shows that the transgenerational effect was maintained for 5 generations of animals in the absence of pathogens.

### Dauer formation under pathogenesis is transmitted to the progeny through the maternal germ line.

**FIG 5:**
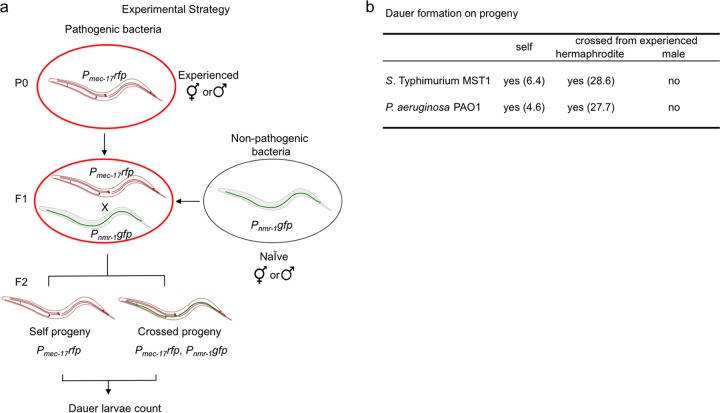
Dauer formation under pathogenesis is transmitted to the progeny through the maternal germ line. (a) Schematic representation of the experimental strategy used for identifying which germ line carries the information to form dauers in the progeny. (b) Dauer formation on self- and crossed progeny.

We next asked whether the dauer information was transmitted through the oocyte or sperm. To answer this question, we exposed *rfp*-marked P0 males or hermaphrodites to pathogens (Fig. 5a, “Experienced”), crossed their F1 progeny with naive hermaphrodites or males expressing *gfp*, and counted the number of dauers in the F2 generation of each cross. If oocytes carry the dauer signal, the crossed progeny of experienced F1 hermaphrodites with naive males should form dauers; however, if sperm cells contain the dauer information, dauer larvae should be found in the crossed progeny of experienced males with naive hermaphrodites (see Materials and Methods for details). On both pathogens, experienced hermaphrodites mated with naive males produced dauers in the second generation that were both crossed and self-progeny (Fig. 5b). Interestingly, the amount of crossed progeny dauers was much higher than the amount of self-progeny dauers. Conversely, experienced males were not able to transmit the dauer information to naive F1 hermaphrodites, since no dauers were found in their crossed progeny (Fig. 5b). This indicates that the maternal germ line carries and transmits the signal(s) to form dauers to the progeny and suggests that a mechanism capable of systemic spreading of information underlies this phenomenon.

### RNA interference is required for defense against bacterial pathogens.

**FIG 6:**
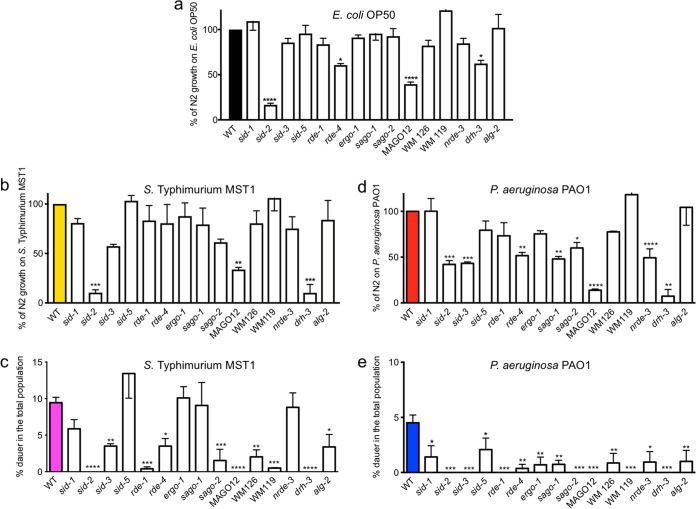
RNAi effectors are needed for pathogen resistance and dauer formation as a transgenerational defense. (a to c) Growth of RNAi mutant animals on *E. coli* OP50 (a), *S*. Typhimurium MST1 (b), and *P. aeruginosa* PAO1 (c) as the percentage of wild-type N2 growth under each condition. (d, e) Dauer formation of RNAi mutant animals on *S*. Typhimurium MST1 (d) and *P. aeruginosa* PAO1 (e). ****, *P* < 0.0001; ***, *P* < 0.001; **, *P* < 0.005; *, *P* < 0.05. Error bars indicate SEM of at least three biological replicas done in triplicates.

RNAi is heritable and systemic and amplifies as the double-stranded RNA (dsRNA) signal enters the organism (21). MicroRNAs (miRNAs) of diverse families have been implicated in the response to the pathogens *P. aeruginosa* PA14 (22, 23) and *Bacillus thuringiensis* strain DB27 (24) in acute infection experiments. We asked whether effectors of the RNAi machinery had a role in defense against the pathogens *P. aeruginosa* PAO1 and *S*. Typhimurium MST1. To answer this question, we quantified the population growth on pathogens of a large number of RNAi-defective mutant strains, excluding those that were nonviable or had severe developmental defects (see Materials and Methods for a complete list of strains used). To evaluate their survival, the population growth of RNAi-defective mutants was compared to the growth of a wild-type strain (strain N2) on *P. aeruginosa* PAO1, *S*. Typhimurium MST1, and *E. coli* OP50 lawns (Table 1). Of note, the growth of environmental RNAi *sid-2* mutants on nonpathogenic *E. coli* OP50 was much lower than that of wild-type animals (Fig. 6a; Table 1). SID-2 is needed to facilitate the import of dsRNA from the intestine (25). We used GFP-tagged *E. coli* OP50 to test whether *sid-2* mutants were colonized by nonpathogenic bacteria compared to the colonization of wild-type animals. *daf-2* mutants, a strain resistant to infection (26) were used as the negative control. Interestingly, *sid-2* animals were heavily colonized by *E. coli* OP50 compared to the colonization of N2 and *daf-2* animals (Fig. S4A). The *E. coli* OP50-GFP colonization of *sid-2* animals was similar to that of *S*. Typhimurium MST1-GFP. *S*. Typhimurium MST1-GFP colonized wild-type nematodes, while *daf-2* animals were refractory to infection (Fig. S4B). This suggests that signaling through SID-2 is necessary for wild-type growth on *E. coli* OP50, which in the absence of SID-2 colonizes the intestine to the same extent as *S*. Typhimurium MST1. The multiple Argonaute mutant strain MAGO12 (see Materials and Methods) also shows a dramatic decrease in growth on *E. coli* OP50 compared to the growth of wild-type animals, reflecting that subsequent steps in RNA processing are also important for growth on nonpathogenic bacteria. Animals growing on pathogens (Fig. 6b and c) responded worse to *P. aeruginosa* PAO1 than to *S*. Typhimurium MST1 exposure, a trend that is also observable in the wild-type nematode strain (Fig. 1a). The growth of more than half of the strains tested was affected on *P. aeruginosa* PAO1, reflecting that the RNAi machinery is necessary for defense against pathogens (Table 1; Fig. 6c).

10.1128/mBio.01234-17.4 FIG S4  Percentages of intestinal colonization of nonpathogenic and pathogenic bacteria in *sid-2* animals. Colonization of *sid-2*, *daf-2*, and wild-type F1 animals exposed to *E. coli* OP50-GFP (A) and *S*. Typhimurium MST1-GFP (B). Download FIG S4, TIF file, 2.8 MB. Copyright © 2017 Palominos et al. 2017 Palominos et al. This content is distributed under the terms of the Creative Commons Attribution 4.0 International license.

**TABLE 1:** Growth and dauer formation of *C. elegans* RNAi mutants in comparison to those of wild-type worms when fed on different bacteria

*C. elegans* strain	Value (%) compared to result for *C. elegans* wild-type strain N2 (set at 100%) when fed on^a^:					
	*E. coli* OP50		*S. Typhimurium* MST1		*P. aeruginosa* PAO1	
	Growth	Dauer formation	Growth	Dauer formation	Growth	Dauer formation
Strains with growth different from N2						
*sid-2*	16.6	0	10.5	**0**	42.1	**0**
MAGO12	39.5	0	33.8	**0**	13.9	**0**
*drh-3*	62.4	0	10.2	**0**	7.4	**0**
*rde-4*	60.6	0			51.6	**8.1**
*sid-3*					43.3	**0**
*sago-1*					48	**16.3**
*sago-2*					60	**0**
*nrde-3*					49.4	**21.1**

Strains with growth like N2						
*sid-1*	NA	0	NA	62.4	NA	**32.8**
*sid-3*	NA	0	NA	**37.2**		
*sid-5*	NA	0	NA	142.9	NA	**46.2**
*rde-1*	NA	0	NA	**4.3**	NA	**0**
*rde-4*			NA	**37.2**		
*ergo-1*	NA	0	NA	107.4	NA	**15.6**
*sago-1*	NA	0	NA	96.5		
*sago-2*	NA	0	NA	16.3		
WM126	NA	0	NA	**21.6**	NA	**19.3**
WM119	NA	0	NA	**5.3**	NA	**0.0**
*alg-2*	NA	0	NA	**36.2**	NA	**22.2**
*nrde-3*			NA	93.6		

a Boldface indicates significant differences in dauer formation compared to the wild-type strain. NA, not applicable.

### RNAi machinery is required for communicating dauer formation response as a defense mechanism.

Systemic RNAi spreads the environmental RNAi signal to the animal cells and tissues. Systemic RNAi pathways involve the triad SID-2/SID-1/SID-3 (25, 27–29) and the endosome-associated protein SID-5 (30). Once dsRNA is inside the cells, the cell-autonomous RNAi machinery, composed of Argonaute proteins, processes and amplifies the signal and produces gene silencing (31). Several RNAi effectors were needed for survival of the wild type in the presence of pathogenic food, while other members of the Argonaute family and RNAi systemic effectors were dispensable for growth on pathogens, probably due to redundancy or more specific roles in the defense process (Fig. 6b and c). We were interested in understanding whether the RNAi machinery is a main player in dauer formation under pathogenesis. Importantly, RNAi is only required for pathogen-induced diapause formation and not for diapause caused by starvation, since all mutants tested formed dauers in the absence of food (Fig. S5).

10.1128/mBio.01234-17.5 FIG S5  Quantification of dauer larvae of RNAi mutants under starvation conditions. Three L4 animals of each genotype were plated on 60-mm plates. Dauer larva count was performed 13 days after animals were plated. Download FIG S5, TIF file, 2.8 MB. Copyright © 2017 Palominos et al. 2017 Palominos et al. This content is distributed under the terms of the Creative Commons Attribution 4.0 International license.

We tested the ability of several RNAi mutants to form dauers in the second generation on *P. aeruginosa* PAO1 and *S*. Typhimurium MST1 lawns. Because low growth also diminishes dauer formation, we reasoned that the more informative strains would be those that grow similarly to wild-type N2 animals on pathogens (Table 1) but fail to produce dauers in their F2 offspring. Except for the *sid-5* mutant, which formed normal amounts of dauers on *S*. Typhimurium MST1, all systemic RNAi mutants were defective in dauer formation on both pathogens (Fig. 6d and f), suggesting that dauer formation under pathogenesis requires the SID-1/SID-3-dependent pathway of dsRNA. RDE-1 and RDE-4, required for exogenous and cell-autonomous RNAi (32, 33), are essential for dauer formation on both pathogens, suggesting that the trigger RNA signal, likely from the pathogen, is recognized by this complex. The multiple-AGO strain WM126, which is resistant to both germ line and somatic RNAi (31), did not form dauers efficiently on either pathogen. Importantly, *sago*-*1* and *sago-2* mutants, which are defective in RNAi amplification, could not be compensated by any other Argonaute, since neither mutant efficiently formed dauers, revealing that the contribution of individual effectors may be limiting for dauer formation under pathogenesis. Interestingly, the functional rescue of muscle RNAi by overexpression of *sago-2* under the *myo-3* muscle-specific promoter (*P_myo-3_sago-2* mutant in strain WM119) (31) could not rescue dauer formation, indicating that other tissues, including the germ line, are key in the signal transmission that leads to the formation of dauers in the progeny. Consistently, the strain with a mutation in *alg-2*, which is important for germ line RNAi, failed to form dauers in both pathogens, also highlighting a role for the endogenous RNAi pathway in dauer formation in response to pathogens. *ergo-1* mutants, which are defective in endogenous RNAi, and mutants with a mutation in *nrde-3*, which is required for nuclear RNAi, failed to form dauers on *P. aeruginosa* PAO1 but could form dauers on *S*. Typhimurium MST1, which may reveal differences in the communication process between these two pathogens and the animal. These results suggest that systemic, exogenous, and endogenous RNAi pathways are required for a productive avoidance strategy against bacterial infection.

### *P. aeruginosa* PAO1 and *S*. Typhimurium MST1 induce nuclear expression of DAF-16 in late F1 larvae and F2 embryos.

**FIG 7:**
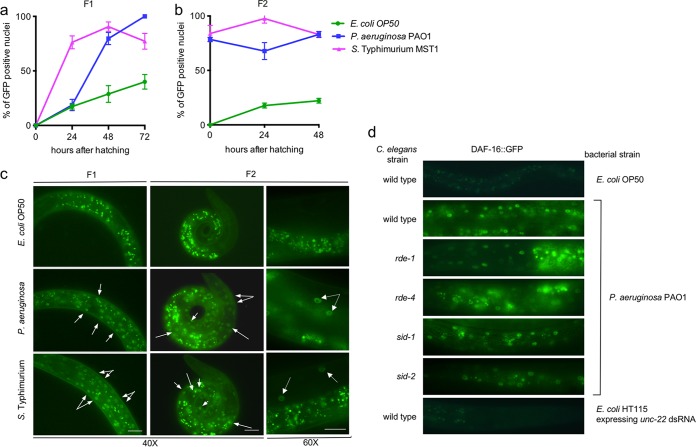
DAF-16 activation begins in the adult animals of the first generation exposed to pathogens. (a, b) Time course of DAF-16::GFP nuclear translocation in *muIs61* F1 (a) and F2 (b) animals feeding on *E. coli* OP50, *P. aeruginosa* PAO1, and *S*. Typhimurium MST1. Points are average results for 90 animals from triplicates. Error bars indicate SEM of at least three biological replicas. (c) Photographs of nuclear expression of DAF-16 in the nuclei of *muIs61* animals as adults in the F1 and young larvae in the F2 generation. Arrows indicate individual GFP expressing nuclei. (d) Nuclear DAF-16::GFP expression of RNAi mutant animals exposed to pathogenic bacteria. Photographs of wild-type and mutant animals expressing DAF-16::GFP after feeding on *P. aeruginosa* PAO1. As controls, wild-type animals were exposed to *E. coli* OP50 and *E. coli* HT115 expressing *unc-22* dsRNA. Scale bars represent 30 μm.

Dauer formation requires DAF-16, since mutations in *daf-16* cause animals to be dauer defective (34). The *daf-16* gene encodes a transcription factor that is negatively regulated by the insulin-signaling (IS) pathway (34). DAF-16 protein normally resides in the cytoplasm but translocates to the nuclei upon exposure to stressful stimuli, such as starvation, heat, and oxidative stress (35). To examine whether DAF-16::GFP translocation was induced in response to pathogens and correlated with the induction of dauer formation, we exposed animals expressing DAF-16::GFP (*muIs61* mutants) (36) to *P. aeruginosa* PAO1 and *S*. Typhimurium MST1 for two generations and examined GFP expression under a fluorescence microscope at 24-h intervals for two generations of progeny. L4 animals (P0) were exposed to bacteria, and their progeny (F1) were collected at the time of hatching, placed on new *P. aeruginosa* PAO1 and *S*. Typhimurium MST1 food, and scored every 24 h for GFP expression in the nuclei for two generations of progeny. DAF-16::GFP translocated to the nuclei in the presence of both pathogens (Fig. 7; Table S1), indicating that they promote a stress response in the animals. Figure 7 shows the percentages of animals with more than 10 GFP-positive nuclei, and Table S1 presents the degrees of GFP expression in animals fed on the three different bacteria (criteria are explained in Materials and Methods). In the F1 animals, both *P. aeruginosa* PAO1 and *S*. Typhimurium MST1 induced DAF-16 nuclear expression significantly at 48 h (80% and 90%, respectively); the induction increased to 100% on *P. aeruginosa* PAO1 and slightly decreased to 77% on *S*. Typhimurium MST1 toward adulthood (Fig. 7a and c). In the F2 animals on both strains of bacteria, the nuclear expression of DAF-16::GFP remained constant, starting at 75% positive nuclei at 24 h and reaching 83% at 72 h (Fig. 7b and c). This suggests that DAF-16 translocation to the nucleus precedes dauer formation. At the time F1 animals show DAF-16 nuclear expression, they are close to adulthood, when animals are developmentally unable to form dauers. This is consistent with dauer formation occurring in the progeny of these animals. Our results show that dauer larva formation induced by pathogens depends on the integration of signals from bacteria, such as their virulence, as well as genetic components of the host, such as translocation of the DAF-16 transcription factor to the nuclei.

10.1128/mBio.01234-17.6 TABLE S1  DAF-16::GFP nuclear expression of two generations of animals exposed to pathogens. Download TABLE S1, DOCX file, 0.01 MB. Copyright © 2017 Palominos et al. 2017 Palominos et al. This content is distributed under the terms of the Creative Commons Attribution 4.0 International license.

We were interested in exploring whether DAF-16 signaling was impaired in RNAi mutants and therefore responsible for their inability to enter diapause under pathogenesis. To answer this question, we generated animals containing mutations in *rde-1*, *rde-4*, *sid-1*, and *sid-2* that also expressed DAF-16::GFP, by crossing the single mutants with the *muIs61* array (36). *P. aeruginosa* PAO1 induces the translocation of DAF-16 to the nucleus in the first progeny of animals exposed to the bacteria, and it remains nuclear throughout the following generation (Fig. 7a). This signaling does not depend on RDE-1, RDE-4, SID-1, or SID-2, since DAF-16::GFP is localized to the nuclei of mutant animals exposed to *P. aeruginosa* PAO1 (Fig. 7d). Also, RNAi by itself does not trigger DAF-16 translocation to the nucleus, since in *unc-22* (RNAi)-treated animals, DAF-16 remained cytosolic (Fig. 7d). These results suggest that DAF-16 activation is mainly part of a stress response to pathogens that is independent of the RNAi pathway.

## DISCUSSION

### Diapause formation as a behavioral transgenerational strategy against pathogens.

Bacterial pathogens can cause disease but also affect behavior. In this work, we show that *C. elegans* worms living on pathogenic bacteria commit a number of their progeny to enter diapause as a transgenerational mechanism to avoid pathogens. Increased dauer formation on a sensitized *daf* mutant background or under high-temperature conditions has been observed in parent animals as an acute response to virulent bacteria (37). Several pathways tightly control diapause entry under classical stress situations, such as starvation and high temperature (6). In this work, we made the novel finding that dauer formation under pathogenesis is a decision transmitted transgenerationally through the maternal germ line. Dauer formation as an avoidance mechanism ensures the survival of the community under circumstances where escaping is not a choice, constituting an evolutionary advantage (5).

### Bacterial trigger of defense strategy.

Our data indicate that dauer formation under pathogenesis requires pathogens of moderate virulence that support growth past the first generation of animals. Neither highly virulent nor apathogenic bacteria trigger dauer formation, establishing a threshold where animals mount a defense response. This is congruent with the idea that the host’s disease threshold depends on the amount of host damage that results from the host-microbe interaction (3). Dauer formation occurred in worms fed on *P. aeruginosa* PAO1, which is moderately virulent, but not in worms fed on *P. aeruginosa* PA14, a highly virulent strain that causes disease in a wide range of organisms (Fig. 1b) (38). Increasing the virulence of PAO1 by P_i_ depletion in the medium also decreased growth and dauer formation (see Fig. S2B and C in the supplemental material).

We show that dauer formation requires a persistent intestinal interaction with the pathogen. F2 embryos reset the dauer decision and commit to a normal developmental program when transferred to nonpathogenic bacteria or UV-killed pathogens. We propose a model where a molecule or RNA signal secreted by pathogens in the intestines of animals is the trigger for dauer formation (Fig. 7). An interesting question that remains to be answered is whether the signal is common to both *P. aeruginosa* PAO1 and *S*. Typhimurium MST1 or whether animals can recognize different bacterial signals or cell envelope components and translate them in a common behavioral response.

### RNAi-dependent defense.

The transgenerational response to pathogens builds up for one generation before it produces a behavioral change in the progeny of animals exposed to pathogens. The maternal germ line can transfer this information when fertilized by sperm from naive males, but sperm cells from experienced animals do not transmit the information to oocytes from naive animals (Fig. 4d). The RNAi machinery mediates systemic and heritable information transfer (21), and thus, it is a good candidate to communicate signals between parent and progeny animals. We show that the environmental RNAi effector SID-2 is required for survival and growth on bacteria that are not usually pathogenic for wild-type worms (Fig. 5a; Table 1). SID-2 is a transmembrane protein expressed in the lumenal membrane of the intestine (25), where it internalizes exogenous dsRNA in a vesicle-driven mechanism that also involves the dsRNA transporter SID-1 (28). *sid-2* animals are heavily colonized by nonpathogenic *E. coli* OP50, as well as by pathogens (Fig. S4). A possibility that remains to be tested is that SID-2 senses and interacts with bacteria in the intestine and communicates the pathogenic status of bacteria through an RNAi mechanism. Given that SID-2 is a dsRNA transporter, it is likely that the bacterial trigger is a dsRNA molecule.

Recently, a link between RNAi and transmission of the environmental stress signal has been established (39). Growth on *bona fide* pathogenic bacteria, implicating a greater challenge to animals, decreased the population numbers of several RNAi mutants with mutations of systemic, exogenous, and endogenous RNAi compared to the growth of wild-type animals (Table 1). These results suggest that several steps along the RNAi pathway are necessary for a response against pathogens in the environment. Consequently, in its natural habitat, where pathogens are plentiful, a percentage of a *C. elegans* population forms the stress-resistant dauer larvae to successfully colonize new food sources (40, 41).

**FIG 8:**
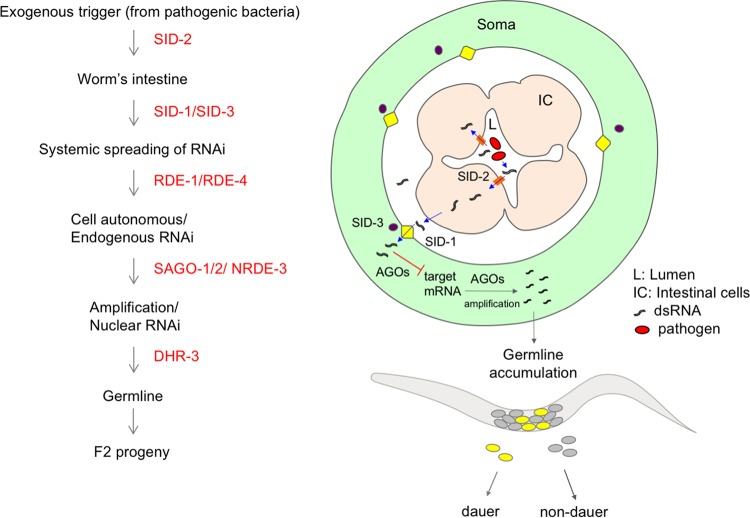
Model of transgenerational RNAi-induced dauer formation in response to pathogens. Pathogenic bacteria in the intestine of *C. elegans* produce a molecule, likely a dsRNA, that constitutes the trigger for the RNAi-dependent transgenerational dauer formation response to pathogenesis. RNAi effectors first transport the dsRNA molecule from the intestine to the soma (SID-2, SID-1, and SID-3) of the animal (in the diagram, all tissues except the germ line are grouped as “soma” for simplicity), and later, the AGO proteins process and amplify the RNA information, which accumulates in the germ line and is inherited by the F2 progeny.

We show in this work that dauer formation is a successful strategy to avoid pathogens and demonstrate that the RNAi machinery is crucial in signaling this behavior to the progeny of infected animals. *P. aeruginosa* PAO1 infection triggers a systemic response in worms that is in part shared with *S*. Typhimurium MST1 and in part unique (unpublished results). Our working model is that the information to form dauers originates, possibly from the bacteria colonizing the intestine, as a bacterial dsRNA trigger that enters the organisms in a SID-2-dependent fashion and spreads to other tissues by a mechanism involving SID-1 and SID-3 for both pathogens, as well as SID-5 for *P. aeruginosa* PAO1. The exogenous dsRNA is initially processed by RDE-1 and RDE-4 to target worm mRNAs and later processed and amplified by the SAGO proteins. This signal is accumulated in the germ line in a process dependent on DHR-3 and ALG-2 (Fig. 8). Small RNAs then target worm transcripts involved in diapause formation in the F2 progeny. Dauer formation in response to *P. aeruginosa* PAO1, but not *S*. Typhimurium MST1, requires NRDE-3-dependent nuclear RNAi in the soma, highlighting that intermediate steps in the defense process are unique for each pathogen. In this work, we propose dauer entry as a strategy for escaping bacterial pathogens which depends on the RNAi machinery and is transmitted to the progeny of infected animals in a transgenerational fashion.

## MATERIALS AND METHODS

### Bacterium and nematode growth.

Bacteria were grown overnight on Luria-Bertani (LB) plates at 37°C from glycerol stocks. The next morning, a large amount of the bacterial lawn was inoculated into LB broth and grown for 6 h with agitation at 250 rpm at 37°C. Three-milliliter amounts of the resulting bacterial culture were seeded onto 90-mm NGM plates and allowed to dry for 36 h before worms were placed on the plates. Wild-type *C. elegans* (strain N2) and mutant strains were grown at 20°C as previously described (42). All nematode strains were grown on *E. coli* strain OP50-1 (resistant to streptomycin) or OP50-GFP (resistant to ampicillin) prior to pathogen exposure. Unless otherwise noted, in all experiments, five wild-type or mutant L4 worms were picked onto each plate seeded with pathogenic and control bacteria, and after 8 days, the total numbers of worms and dauer larvae were counted.

### Bacterial strains.

We used the following wild-type bacterial strains: *E. coli* OP50-1, *E. coli* OP50-GFP, *Salmonella enterica* serovar Typhi Ty2 (ATCC 700931), *S. enterica* serovar Typhimurium MST1, *S. enterica* serovar Typhimurium 14028 (MST1 strain containing the GFP-expressing plasmid 14028 [ATCC 14028], called MST1-GFP throughout the article), *Pseudomonas aeruginosa* PA14, *P. aeruginosa* PAO1 (ATCC 15692), and *P. aeruginosa* PAO1-GFP (containing the plasmid pSMC2) (43). We used the following *P. aeruginosa* PAO1 avirulent strains: PW3598 (*lasR*-CO1::IS*lacZ*/*hah*), PW9826 (derived from PAO1 with the insertion of mini-Tn*5*-Tet^r^ in *ppk1* [PA5242]); and PW5085 (lacZbp03q1H07) with a mutation in *pvdS* (PA2426).

### *C. elegans* strains.

We used the following *C. elegans* mutant strains: CB1370 [*daf-2(e1370)III*] (44), NL3321 [*sid-1(pk3321)V*] (45), HC271 [*sid-2(qt42)III*] (25), VC787 [*sid-3(ok973)X*] (*C. elegans* Reverse Genetics Core, Vancouver, Canada), FX04328 [*sid-5(tm4328)*] (30), WM27 [*rde-1(ne219)V*] (32), WM49 [*rde-4(ne301)III*] (33), W158 [*ergo*-1*(tm1860)V*] (46), WM53 [*alg-2(ok304)II*] (*C. elegans* Gene Knockout Consortium), YY158 [*nrde-3(gg66)X*] (47), WM206 [*dhr-3(ne4253)I*] (48), WM119 [*sago-2(tm894) ppw-1(tm914)I*; C06A1.4*(tm887)* F58G1.1*(tm1019)II*; M03D4.6*(tm1144) IV*; *sago-1(tm1195)V*; *neIs10X*] (31), WM126 [*sago-2(tm894) ppw-1(tm914)I*; C06A1.4*(tm887)* F58G1.1*(tm1019)II*; M03D4.6*(tm1144)IV*; *sago-1(tm1195)V*] (31), WM160 [*sago-1(tm1195)V*] (31), WM154 [*sago*-2*(tm894)I*] (31), WM191 (called the MAGO12 mutant herein) [s*ago-2(tm894)I*; *ppw-1(tm914)I*; *ppw-2(tm110)I*; F55A12.1*(tm2686)I*; R06C7.1*(tm1414)I*; Y49F6.1*(tm1127)II*; ZK1248.7*(tm1113)II*; F58G1.1*(tm1019)II*; C16C10.3*(tm1200)III*; K12B6.1*(tm1195)III*; T22H9.3*(tm1186)V*; R04A9.2*(tm1116)X*] (48), CF1139 [*daf-16(mu86)I*, *muIs61*] (36), TU2769 [*uIs31(P_mec-17_mec-17*::*gfp)*] (49), and WCH33 [*deg-1*; *akIs31(P_nmr-1_nmr-1*::*gfp)*] (50), Additionally, we generated strains expressing *muIs61* with mutations in *rde-1* (WCH29), *rde-4* (WCH30), *sid-1* (WCH20), and *sid-2* (WCH31).

### Quantification of dauer larvae. (i) Dauer formation on pathogens.

The entire worm population on each plate was collected in 1 ml of M9 medium. This initial stock was diluted 1:10 in M9 medium to count the total population or in 1% SDS to count the amount of dauers after 20 to 30 min of incubation (13). Ten microliters of each dilution was used to count the total population and the dauer larvae, respectively, under a Nikon SMZ745 stereomicroscope. Each condition was scored 3 times, and the amounts of dauers were plotted as percentages of the total populations of animals.

### (ii) Dauer formation under starvation conditions.

Three L4 animals each were picked onto 60-mm plates seeded with *E. coli* OP50-1. Eight days after the food was exhausted, the total populations and dauer larvae were counted.

### (iii) Dauer formation in successive generations.

Forty wild-type L4 worms (P0) were transferred to 90-mm plates seeded with *E. coli* OP50-1 or *S. enterica* serovar Typhimurium MST1 and 60 L4 worms to *P. aeruginosa* PAO1 lawns. At the fourth day, we picked 40 L4 worms from *E. coli* OP50-1 and *S*. Typhimurium MST1 plates or 60 L4 worms from *P. aeruginosa* PAO1 plates and transferred them to new plates with the same bacteria. Worms were allowed to grow for 5 days on *E. coli* OP50-1 and *S*. Typhimurium MST1 and for 6 days on *P. aeruginosa* PAO1. This procedure was done in triplicates and repeated for five consecutive generations of the same starting population.

### (iv) Dauer formation of F2 animals shifted between different bacterial strains.

Ten wild-type L4 worms (P0) were transferred to *E. coli* OP50-1 and *S*. Typhimurium MST1 and 15 to *P. aeruginosa* PAO1 lawns seeded in 90-mm plates. After 4 days, worms were collected with M9 buffer and their embryos were extracted by sodium hypochlorite treatment (15% hypochlorite solution, 0.1 M NaOH). F2 embryos from plates with each of the three bacteria were pelleted by centrifugation (2,000 rpm for 2 min), divided, and transferred to new plates seeded with *E. coli* OP50-1, *S*. Typhimurium MST1, and *P. aeruginosa* PAO1 live lawns. In addition, F2 embryos from worms grown on *S*. Typhimurium MST1 and *P. aeruginosa* PAO1 were transferred to new plates of UV-killed *S*. Typhimurium MST1 and *P. aeruginosa*, respectively. All experiments were done in triplicates of at least three biological replicas.

### Quantification of pharyngeal pumping.

Thirty to 40 synchronized newly hatched L1 animals were collected using a mouth pipette (51) containing M9 supplemented with 4.5 µl/ml chloride to eliminate *E. coli* OP50 cells and transferred to plates seeded with *E. coli* OP50, *S*. Typhimurium MST1, and *P. aeruginosa* PAO1 in triplicates of each. After 72 h, we measured the pharyngeal contractions per minute by using a manual counter under a Nikon SMZ745 stereomicroscope. Thirty worms were counted per plate per condition.

### Transgenerational dauer formation.

We designed a protocol where control animals were maintained on *E. coli* OP50 for six generations (Fig. 4, “always on *E. coli* OP50”), and others were exposed to *P. aeruginosa* for two generations and passed as F3 embryos to *E. coli* OP50 until the F5 generation (Fig. 4, “subject to changes in food”). F5 embryos were then transferred to *P. aeruginosa* PAO1 plates again until the F6 generation. Importantly, we had six plates for each condition; three were used for dauer counts and three for hypochlorite treatment of adults. We performed dauer counts on every generation regardless of the bacteria animals were on. In detail, we exposed 5 P0 animals to pathogenic *P. aeruginosa* PAO1 or *E. coli* OP50 on 90-mm plates. Sixty L4 larvae from the F1 generation were then transferred using a platinum worm pick to plates seeded with either *E. coli* OP50 or *P. aeruginosa* PAO1. F2 adults were treated with sodium hypochlorite, and the embryos were passed to new plates of *E. coli* OP50. The same treatment was done with the F3 and the F4 generations. F5 embryos from bleached F4 adults were passed to pathogens, and their F6 progeny as well, using the same treatments. To count the number of generations the transgenerational effect is maintained, F2 embryos, grown to that point on *P. aeruginosa* PAO1, were transferred to *E. coli* OP50 lawns. A fraction of every subsequent generation was then transferred to nonpathogenic *E. coli* OP50 and another fraction was transferred to pathogens to test whether animals retained the ability to form dauers (Fig. 4c). For each passage, adult hermaphrodites were treated with sodium hypochlorite, and a fraction of the embryos were transferred to new *E. coli* OP50 lawns and another fraction to *P. aeruginosa* PAO1 lawns. Embryos on *P. aeruginosa* PAO1 were allowed to grow for 72 h before treating the entire plate with 1% SDS for the quantification of dauers. All experiments were done in three biological replicates of three technical replicates each.

### Developmental time course of dauer formation on pathogens.

Five L4 worms each were grown on *E. coli* OP50-1, *P. aeruginosa* PAO1, or *S*. Typhimurium MST1 for 2 days. We synchronized the worms by collecting newly hatched L1 larvae with a mouth pipette 2 h after washing off a plate with mixed-stage worms. Between 90 and 120 newly hatched L1 larvae were transferred to the same bacteria where their P0 parents had grown. We followed the development of F1 and F2 worms grown on *E. coli* OP50-1, *P. aeruginosa* PAO1, or *S*. Typhimurium MST1 every 24 h for 5 days under a Nikon Eclipse Ti-5 fluorescence microscope with ×40 or ×60 magnification. Worms with thickened cuticle, L2 appearance, gonadal arrest, and lack of pharyngeal pumping were classified as dauerlike. The percentage of worms in each larval and adult stage was calculated as the percentage of the total population of animals. Experiments were done in triplicates for each time point.

### Germ line inheritance.

To test whether the information to form dauers in the F2 generation is transmitted to the progeny by the oocyte or sperm, we exposed 20 L4 hermaphrodites expressing a red fluorescent protein (RFP) marker (*P_mec-17_rfp*) to *E. coli* OP50-1, *Salmonella* Typhimurium MST1, or *P. aeruginosa* PAO1 and allowed them to lay eggs for 2 days. Newly hatched F1 larvae were collected with M9 and transferred to new plates with the same bacteria their P0 parents grew on. After 2 days, 20 L4 (F1) hermaphrodites expressing *P_mec-17_rfp* were crossed with 40 naive males expressing *P_nmr-1_nmr-1*::*gfp* (grown on *E. coli* OP50-1). After 4 days, we counted the total population and dauers of each color (*P_mec-17_rfp* self-progeny and *P_mec-17_rfp; P*_*nmr-1*_ *nmr-1*::*gfp* crossed progeny). Conversely, 20 *P_nmr-1_nmr-1*::*gfp* males were grown with *P_nmr-1_nmr-1*::*gfp* hermaphrodites on *E. coli* OP50-1, *Salmonella enterica* serovar Typhimurium MST1, or *P. aeruginosa* PAO1. After 4 days, we crossed 40 *P_nmr-1_nmr-1*::*gfp* L4 experienced males with 20 *P_mec-17_rfp* L4 naive hermaphrodites. Four days later, the total population and dauers of each color were counted. All experiments were done in triplicates, and total worms and dauers were quantified under a Nikon Eclipse Ni fluorescence microscope.

### Depletion of inorganic phosphate from medium.

To increase the virulence of *P. aeruginosa* PAO1, we removed inorganic phosphate from the NGM medium (15). Five L4 larvae were picked into low-P_i_ *P. aeruginosa* PAO1 plates, and after 8 days, total population and dauer quantification was done as described above.

### Siderophore quantification.

To semiquantitatively determine the overall production of siderophores, a chrome-azurol S (CAS) agar diffusion assay was used as previously described (52). CAS agar plates were punched to create small holes that were filled with 100 μl of *P. aeruginosa* PAO1 culture supernatant. The plates were incubated overnight at 37°C. We measured the yellow or orange haloes formed around each hole as total siderophore activity. Deferoxamine mesylate (Desferal; Sigma-Aldrich, USA) was used as an iron-chelating standard.

### Microscopy.

For observation, worms were mounted on 2% agarose pads, paralyzed with 1 mM hydrochloride (Sigma-Aldrich), and visualized under a Nikon Eclipse Ti-5 fluorescence microscope with ×40 or ×60 magnification under Nomarski optics or fluorescence. All images were analyzed and edited with ImageJ (version 1.46).

### Quantification of bacteria in the intestines of *C. elegans* worms*.*

Twenty to 30 L4 animals were individually picked into an Eppendorf tube containing M9 buffer with 25 mM levamisole hydrochloride (Sigma-Aldrich) to cause paralysis and stop pharyngeal pumping (53). The animals were then washed three times with M9 containing 1 mg/ml gentamicin (Sigma-Aldrich) and 1 mg/ml ampicillin (Sigma-Aldrich). After the third wash, the animals were incubated once more with the antibiotic mixture for 1 h. To eliminate the antibiotic, the animals were washed three more times with M9 containing 25 mM levamisole. Each worm pellet was lysed with an individual pestle, and the resulting lysate was serially diluted 1:10 seven times in M9. Amounts of 200 μl of dilutions 5, 6, and 7 were individually plated on LB with streptomycin (to select *E. coli* OP50), with ampicillin (to select *Salmonella*), and without antibiotics for *P. aeruginosa* PAO1. The plated dilutions were incubated overnight at 37°C. The amount of CFUs was calculated using the following formula: CFU per worm = [(colonies per plate/dilution factor) × plated volume]/number of worms.

### UV killing of bacteria.

Ninety-millimeter NGM plates seeded with 3 ml of *E. coli* OP50-GFP, *P. aeruginosa* PAO1, and *S*. Typhimurium MST1-GFP were irradiated for 20 min in a UV transilluminator to kill bacteria. Five L4 worms were picked onto each plate, and after 8 days, we quantified total worms and dauers. To show that UV killed bacteria, we streaked UV-treated bacteria onto LB plates and allowed them to grow overnight at 37°C. Twenty minutes was sufficient to kill all bacteria on the plate.

### Supplementation of *E. coli* OP50 pellet with pathogen supernatant.

Twenty-milliliter amounts of *E. coli* OP50-1, *P. aeruginosa* PAO1, and *S*. Typhimurium MST1 cultures grown overnight on LB broth at 37°C were pelleted by centrifugation at 14,000 rpm for 15 min. Each pathogen supernatant was filtered twice with syringe-driven filters (30 mm by 0.2 μm; Biofilter) and added to a pellet of *E. coli* OP50-1. Three-milliliter amounts of the mixtures were inoculated onto 90-mm plates. Five N2 worms were picked onto each plate, and 8 days later, the total population and dauers were counted. To ensure that the supernatants contained no living bacteria, 100-μl amounts of the pathogen supernatants were seeded onto LB plates and incubated overnight at 37°C.

### Time course of DAF-16::GFP translocation following pathogen exposure.

To generate a temporal curve of DAF-16::GFP nuclear translocation, we picked five CF1139 [*daf-16(mu86); muIs61*] L4 worms (P0) onto *E. coli* OP50-1, *P. aeruginosa* PAO1, or *S. enterica* serovar Typhimurium MST1 and allowed them to lay eggs for 2 days. Synchronized L1 larvae were collected with a mouth pipette 2 h after washing off a plate with mixed-stage worms. Forty-five newly hatched worms (F1) were gently transferred with a mouth pipette to *E. coli* OP50-1, *P. aeruginosa* PAO1, or *S*. Typhimurium MST1 bacterial lawns. We observed the F1 animals for 72 h and their progeny (F2) for 48 h and scored their GFP expression every 24 hours as weak, medium, and high according to the number of fluorescent nuclei in each individual. For the quantification of the percentages of DAF-16::GFP translocation shown in Fig. 7, we considered animals with more than 10 GFP-positive nuclei (medium and high fluorescence). Table S1 depicts the degree of GFP expression in worms fed on the three strains of bacteria, including those with weak expression, which is not considered in Fig. 3. GFP expression in worms with up to 10 GFP-positive nuclei at 150 gain and 1.5-ms exposure was considered weak, up to 30 was considered medium, and above 30 was considered high. Experiments were done in triplicates for each time point.

### DAF-16::GFP translocation to the nuclei in RNAi mutants.

Synchronized N2 and RNAi mutant L1 larvae were collected with a mouth pipette 2 h after washing off a plate with mixed-stage worms. Forty-five newly hatched worms (F1) were gently transferred with a mouth pipette to bacterial lawns of *E. coli* OP50-1, *E. coli* HT115 expressing *unc-22* dsRNA, or *P. aeruginosa* PAO1. To observe GFP translocation to the nuclei, we scored the F1 worms 72 hours after hatching under each condition. Experiments were done in triplicates for each time point.

### Quantification of intestinal fluorescent bacteria.

Fluorescent *E. coli* OP50-GFP and *S*. Typhimurium MST1-GFP were streaked on LB-ampicillin plates (50 mg/ml in 30 ml) and grown overnight at 37°C. The next day, one bacterial colony was picked and grown on liquid LB at 37°C for 6 h. One hundred fifty milliliters of each bacterial culture was seeded onto 60-mm NGM agar plates. Twenty L4-stage N2, *sid-2*, or *daf-2* animals were picked onto *E. coli* OP50-GFP or *S*. Typhimurium MST1-GFP lawns. We followed the intestinal fluorescence produced by bacteria every 24 h for two generations of animals. We classified the colonization as undetectable when no fluorescence was seen in the intestine or discrete bacteria appeared only in the pharynx, partial when one-third of the intestine contained fluorescent bacteria, and full when all of the intestine was fluorescent. At each time point, we scored 15 to 30 worms.

### Biological and technical replicates.

Each of the experiments was performed in three technical triplicates and at least three biological replicates. Each technical replicate was read three different times. We defined biological replicates as experiments made on different days, containing triplicates of each condition, and technical replicates as triplicates of the same condition on the same day. For example, in an experiment comparing dauer formation on pathogenic versus nonpathogenic bacteria, we used three plates (technical replicates) per bacterium, plated a fixed number of worms in them, and counted the population and dauers after 8 days, three times per replicate (as described above). The average of the three reads of each triplicate was considered one count. Each experiment had three technical replicates that were in turn averaged to constitute one of the data points of each figure. The data were collected and processed as a single technical replicate (the average of three counts of the same plate), and its mean was used as a single biological replicate. Each figure presents the results of at least three experiments (biological replicates) performed as explained above. All the biological replicates were performed at intervals of 1 day to 1 week from each other.

### Criteria for data exclusion.

We excluded data from entire experiments when there was contamination with unwanted bacteria or fungi on the worm plates. We also excluded data from experiments where bacteria had been almost completely consumed.

### Sample size.

Each experiment started with 5 L4 parental worms on each technical triplicate. After the second generation, the number of worms on each plate ranged from 20,000 to 40,000 on *E. coli* OP50, 11,000 to 20,000 on *P. aeruginosa* PAO1, and 20,000 to 30,000 on *S*. Typhimurium MST1.

### Statistical evaluation.

Statistical evaluation was performed using one- or two-way analysis of variance (ANOVA), with *post hoc* tests or two-tailed *t* test when appropriate. The results of all tests are detailed in Data Set S2.

10.1128/mBio.01234-17.7 DATA SET S1  Excel file containing all the raw data from the paper. Sheet 1 contains all data used for the calculations, and the rest of the sheets, named after the figures, contain the averaged data for each individual figure. Download DATA SET S1, XLSX file, 0.1 MB. Copyright © 2017 Palominos et al. 2017 Palominos et al. This content is distributed under the terms of the Creative Commons Attribution 4.0 International license.

10.1128/mBio.01234-17.8 DATA SET S2  Excel file containing all statistical analysis for the paper. Download DATA SET S2, XLSX file, 0.04 MB. Copyright © 2017 Palominos et al. 2017 Palominos et al. This content is distributed under the terms of the Creative Commons Attribution 4.0 International license.

10.1128/mBio.01234-17.11 MOVIE S3  *C. elegans* culture of F2 animals feeding on *E. coli* prior to treatment with 1% SDS to quantify dauer larvae formation. Please note that all plates have bacteria in them. Download MOVIE S3, MOV file, 15.6 MB. Copyright © 2017 Palominos et al. 2017 Palominos et al. This content is distributed under the terms of the Creative Commons Attribution 4.0 International license.
