# Supplementary figures and images for: Transgenerational Diapause as an Avoidance Strategy against Bacterial Pathogens in Caenorhabditis elegans
Source: mBio. 2017 Oct 10;8(5):e01234-17. doi: 10.1128/mBio.01234-17 (PMC5635688; doi:10.1128/mBio.01234-17)

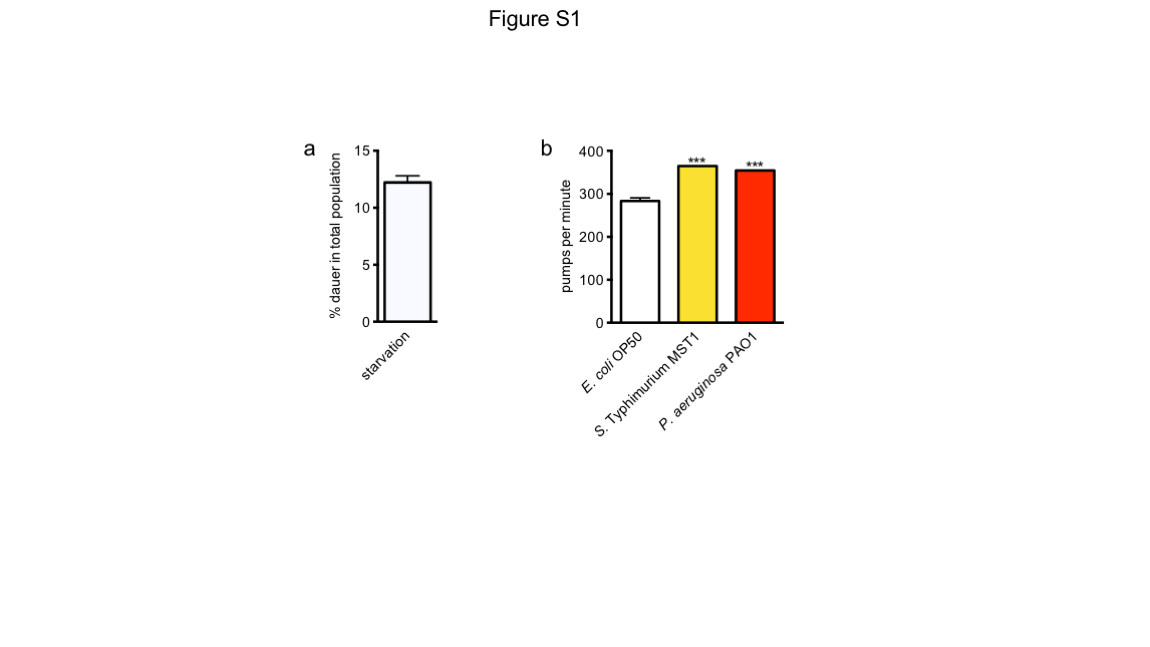

Supplement: FIG S1 [file mbo005173513sf1.tif]

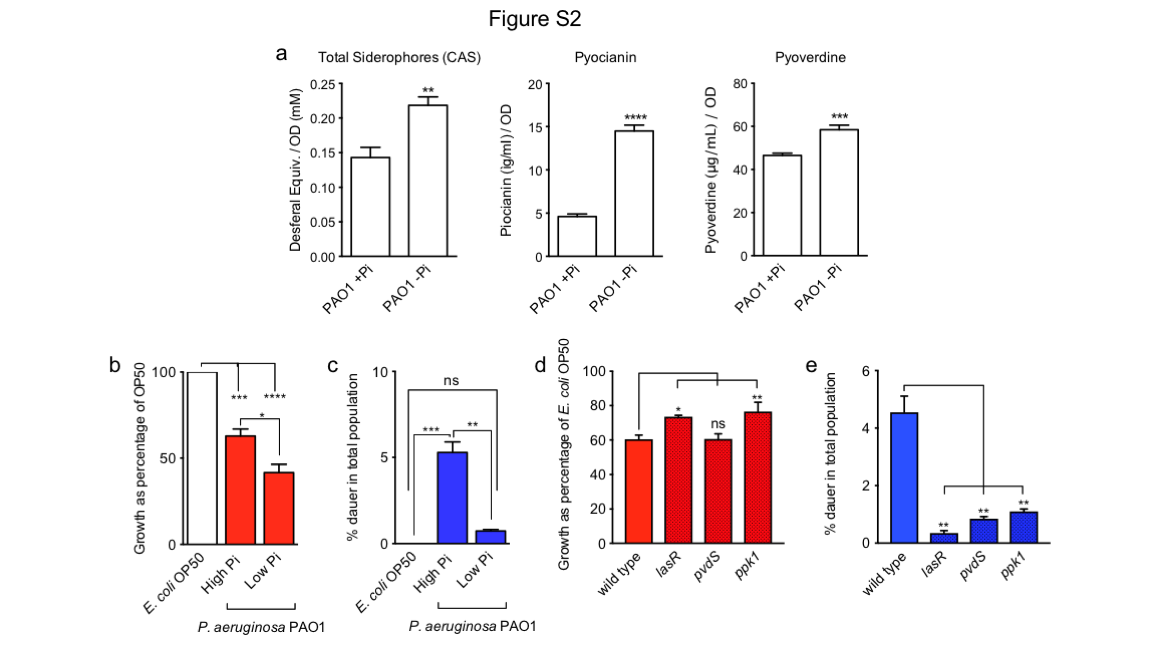

Supplement: FIG S2 [file mbo005173513sf2.tif]

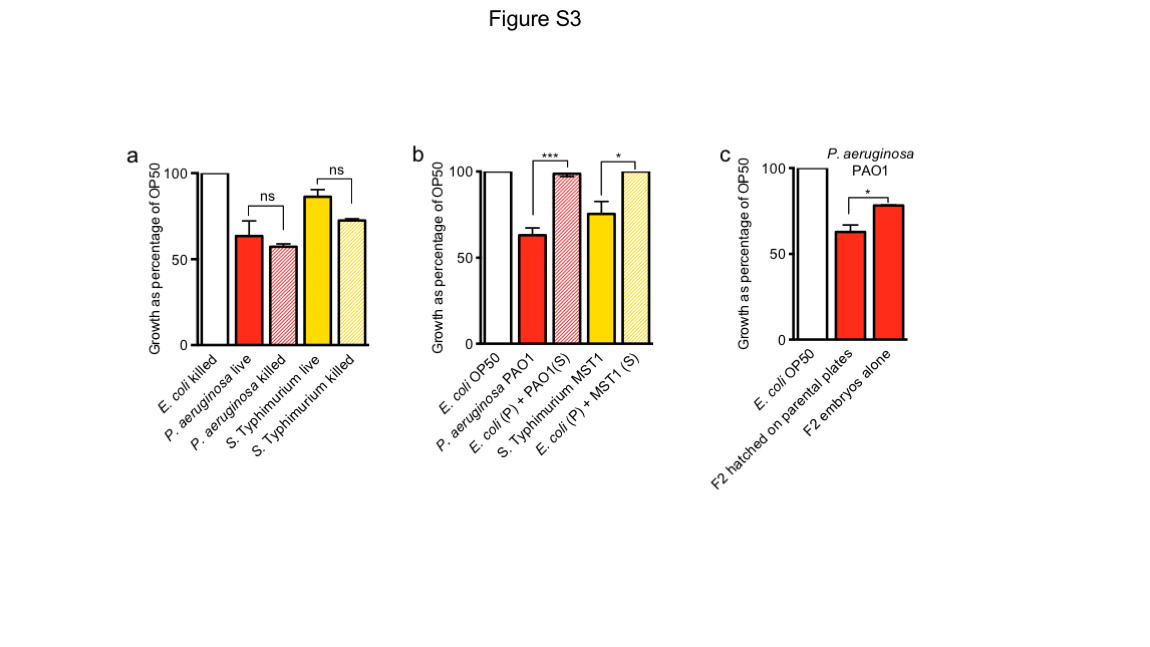

Supplement: FIG S3 [file mbo005173513sf3.tif]

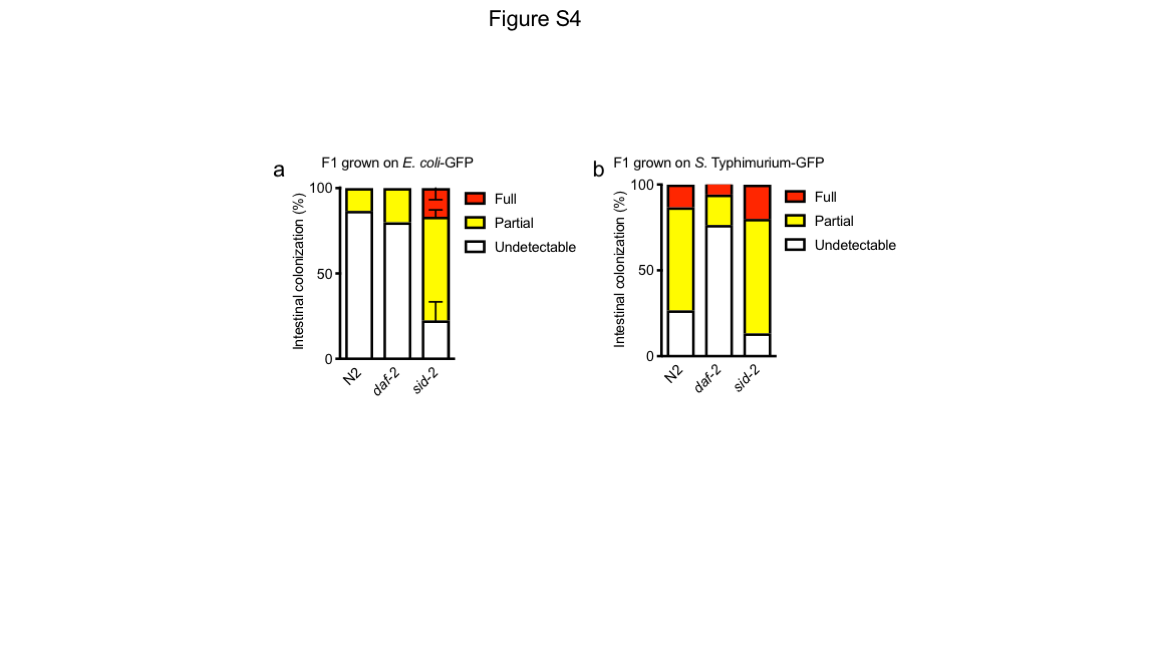

Supplement: FIG S4 [file mbo005173513sf4.tif]

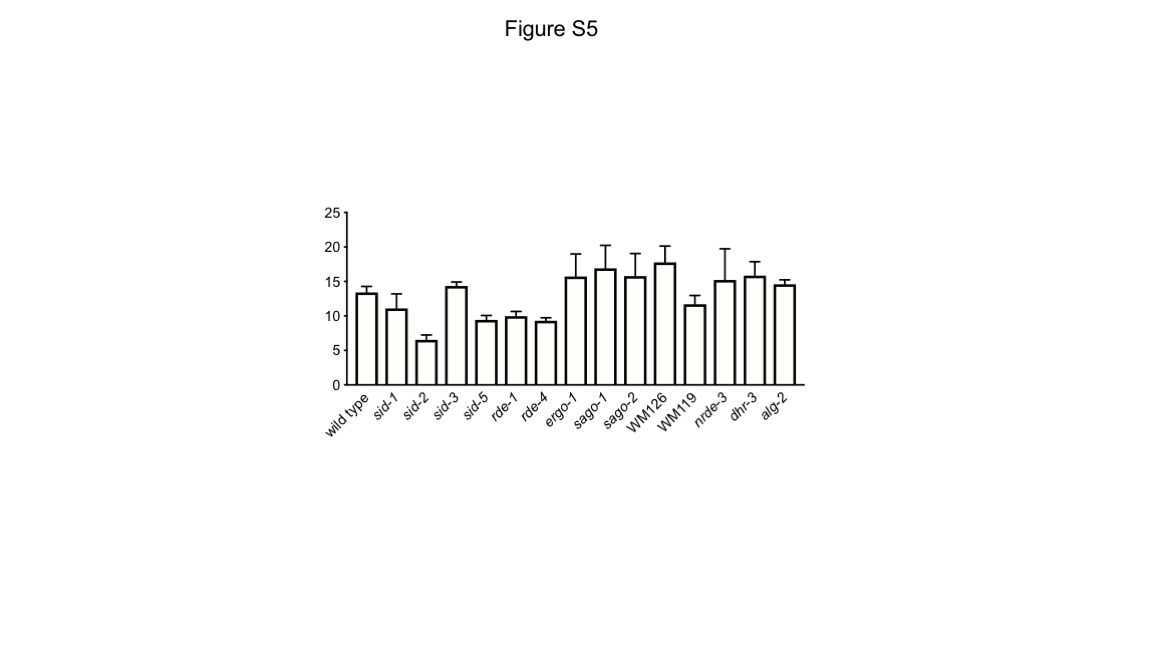

Supplement: FIG S5 [file mbo005173513sf5.tif]
