# Supplementary material for: Transgenerational Diapause as an Avoidance Strategy against Bacterial Pathogens in Caenorhabditis elegans
Source: mBio. 2017 Oct 10;8(5):e01234-17. doi: 10.1128/mBio.01234-17 (PMC5635688; doi:10.1128/mBio.01234-17)
Supplement: TABLE S1 [file mbo005173513st1.docx]

Table S1. DAF-16::GFP nuclear expression of two generations of animals exposed to pathogens

|  | F1 | | | | F2 | | | |  |
| --- | --- | --- | --- | --- | --- | --- | --- | --- | --- |
| Bacterial strains | Time after hatching (hrs) | | | | | | | |  |
|  | 0 | 24 | 48 | 72 | | 0 | 24 | 48 | |
| *E. coli* OP50 | - | * | * | ** | | - | * | * | |
| *P. aeruginosa* PAO1 | - | * | ** | *** | | *** | ** | ** | |
| *S.* Typhimurium MST1 | - | ** | *** | *** | | *** | *** | ** | |

Legend GFP positive nuclei

| - | less than 10 |
| --- | --- |
| * | weak, up to 10 |
| ** | medium, up to 30 |
| *** | high, more than 30 |
